# A217 TREATMENT ADHERENCE OF CHRONIC HEPATITIS B PATIENTS WITH HEPATOCELLULAR CARCINOMA FROM THE CANHEPB NETWORK

**DOI:** 10.1093/jcag/gwab049.216

**Published:** 2022-02-21

**Authors:** S H Rahman, Y Scharr, J Jeyaparan, A Manko, C S Coffin, S E Congly, A Ramji, S Fung, C Cooper, M Ma, R Bailey, G Minuk, A Wong, K Doucette, M Elkhashab, P Wong, M Brahmania

**Affiliations:** 1 internal medicine, Western University, London, ON, Canada; 2 Liver Unit, Division of Gastroenterology, Department of Medicine, University of Calgary Cumming School of Medicine, Calgary, AB, Canada; 3 Gastrointestinal Research Institute, Vancouver, BC, Canada; 4 University of Alberta, Edmonton, AB, Canada; 5 Toronto Liver Centre, Toronto, ON, Canada; 6 Toronto General Hospital, Toronto, ON, Canada; 7 Medicine, University of Calgary, Calgary, AB, Canada; 8 Biological sciences, University of Calgary, Calgary, AB, Canada; 9 University of Manitoba, Winnipeg, MB, Canada; 10 University of Ottawa Faculty of Medicine, Ottawa, ON, Canada; 11 Royal Alexandra Hospital, Edmonton, AB, Canada; 12 Gastroenterology, McGill University, Brossard, QC, Canada; 13 University of Saskatchewan, Saskatoon, SK, Canada

## Abstract

**Background:**

Chronic hepatitis B (CHB) is the most common cause of hepatocellular carcinoma (HCC) worldwide.

**Aims:**

The primary aim of this study is to explore the degree of treatment adherence to the American Association For The Study of Liver Disease (AASLD) HCC treatment guidelines for patients with CHB-HCC.

**Methods:**

This is a retrospective, cross-sectional study of available data (2005–2020) in patients mono-infected with CHB collected from the Canadian HBV Network; a national consortium across 8 Canadian provinces. We analyzed data using descriptive statistics along with parametric and nonparametric statistical methods with a significance level of p < 0.05.

**Results:**

Of the 6500 patients, 132 (2.0%) patients met inclusion criteria. The median age was 64 (IQR: 53.5- 71.5) with 101 (76%) being male. The median ALT was 40 (IQR: 26–59.5) and the median tumor number was 1(IQR: 1- 2) with a median tumor size of 2.6 cm (IQR: 1.9- 4.5). 98 (74.5%) patients were HBeAg negative with a median viral load of 3.8 logs (IQR 1.9 – 5.8). 58 (43%) patients had cirrhosis at diagnosis. 36% of patients were diagnosed with HCC on their first screening imaging whereas 39% were found to have HCC on repeated surveillance imaging. 116 (87.9%) were on treatment at the time of diagnosis or after (70 (60.3%) NA and 46 (39%) Combination therapy with double NA or NA plus interferon). Out of the 132 patients, BCLC stage 0, A, B, and C represented 30 (23%), 42 (32%), 17 (13%), and 5 (4%) patients, respectively, with 38 (28%) patients with unknown BCLC stage. The overall adherence to AASLD guidelines was 61%. The HCC treatment adherence rate for patients with BCLC stage 0, A, B were 63%, 97.5%, and 23.5%, respectively. BCLC stages C and D did not have a sufficient sample size for analysis. The adherence rate ranged from 53% (Eastern Canada) to 71% (Western Canada) across Canada.

**Conclusions:**

In this retrospective nationwide cohort study of patients with CHB-related HCC, the overall treatment adherence rate to AASLD guidelines was low with notable regional differences. Further analysis will determine the cause of regional differences.

**Funding Agencies:**

None

